# Return to work after surgical clipping versus endovascular treatment for ruptured intracranial aneurysms – A nationwide registry-based study

**DOI:** 10.1371/journal.pone.0278528

**Published:** 2022-12-13

**Authors:** Paulina Majewska, Marie Søfteland Sandvei, Sasha Gulati, Tomm B. Müller, Karen Walseth Hara, Pål Richard Romundstad, Ole Solheim

**Affiliations:** 1 Department of Neurosurgery, St. Olav’s University Hospital, Trondheim, Norway; 2 Department of Neuromedicine and Movement Science, NTNU, Trondheim, Norway; 3 Department of Clinical and Molecular Medicine, NTNU, Trondheim, Norway; 4 The Cancer Clinic, St. Olav’s University Hospital, Trondheim, Norway; 5 Department of Public Health and Nursing, NTNU, Trondheim, Norway; 6 The Norwegian Labour and Welfare Administration for Trøndelag, Trondheim, Norway; 7 The National Competence Service for Complex Symptom Disorders, St. Olav’s University Hospital, Trondheim, Norway; NIHR Leicester Biomedical Research Centre, UNITED KINGDOM

## Abstract

**Objectives:**

The aim of this study was to assess return to work following aneurysmal subarachnoid haemorrhage (SAH) and compare working status after open surgical clipping and endovascular treatment.

**Methods:**

This nationwide registry-based study included all adult patients in working age treated for a ruptured intracranial aneurysm in Norway between 2008 and 2018 who had a record of sickness leave on the day of treatment. Data from The Norwegian Patient Registry and The Norwegian Labour and Welfare Administration were linked on an individual level. Daily sickness and disability benefits recipiency one year preoperatively to one year postoperatively was analysed. Return to work after endovascular treatment and surgical clipping was compared.

**Results:**

183 patients were included in the study. Among patients who worked at one year preoperatively, 57% had returned to work one year after treatment. Mean number of days from treatment to the first day back at work in a continuous 3-month working period was 298 (95% CI: 276–321) vs. 319 (95% CI: 299–339) for patients who underwent endovascular treatment compared to patients treated with clipping (*p* = 0.365). Older patients were less likely to return to work after treatment (hazard ratio 0.977 per year of age, 95% CI 0.956–1.000, *p* = 0.046). There was no significant association between return to work and patient sex or location of the aneurysm.

**Conclusions:**

Aneurysmal SAH profoundly affects patient working status. This study found no significant difference in time to return to work after treatment between patients treated with endovascular techniques compared to patients undergoing open surgery.

## Introduction

The estimated prevalence of intracranial aneurysms (IAs) is around 2% [1, 2]. While the average annual risk of rupture is below 1% [3], 37% of patients die within a year after aneurysmal subarachnoid haemorrhage (aSAH) [4].

The International subarachnoid aneurysm trial (ISAT) that recruited patients from 1997–2002 showed better functional results, but somewhat more re-bleedings after endovascular coiling than after open surgical clipping for ruptured intracranial aneurysms [5]. Although treatment, especially the endovascular techniques, have developed much since, the choice of surgical clipping versus endovascular treatment can still be controversial, and there are large treatment variations across centers [6].

Morbidity from aSAH and its treatment has traditionally been measured as impaired neurological function. However, patients may suffer from fatigue, anxiety, depression, post-treatment pain, cognitive challenges, or other symptoms that are difficult to assess using gross neurological function scales. Especially in patients with good outcomes, the total level of function may instead be reflected in the ability to return to work after treatment [7]. The aim of this study was to assess return to work following aSAH and compare working status after open surgical clipping to endovascular treatment.

## Materials and methods

This is a nationwide registry-based study. All adult patients (≥ 18 years old) treated for a ruptured IA in Norway between 2008 and 2018 were identified from the Norwegian Patient Registry (NPR) [8]. Coding of neurological and neurosurgical diagnoses in NPR is of high quality, and data from this registry can safely be used for medical research purposes [9]. All included patients had a Norwegian national security number, thus international patients without permanent residence permit treated in Norway were excluded. Case identification was done based on ICD-10 diagnosis code I60.0-I60.9 [10] and the procedural codes for treatment according to the Nomesco Classification of Surgical Procedures [11] (NCSP codes AAC00–AAC15 [surgery] and AAL00 [endovascular treatment until 2015] and AAY00B [endovascular treatment from 2016]). Patient age at treatment and dates of diagnosis and treatment were retrieved from the same registry. History of sickness absence (sickness and disability benefits) in the period one year before and after treatment was retrieved from The Norwegian Labour and Welfare Administration (NAV) records [12] for all patients. This national registry holds data on all sickness absence certified by a doctor and all disability benefits received by individuals with a Norwegian national security number. Dates of death and retirement were also retrieved from the same registry. NPR and NAV data were linked on the individual level using a national security number that is used as patient identification number in all contacts with health care and social care in Norway.

The retirement age in Norway is 67 years, for both women and men. Therefore, patients older than 66 years at the time of treatment were excluded (as older individuals were most likely going to retire regardless of treatment for aSAH during the one year observation period). In cases where patients were treated multiple times for intracranial aneurysm outside the observation period (i.e. multiple episodes with aneurysm treatment more than a year apart), only the first treatment for a ruptured IA was included in the analyses. All patients included in the analyses had to have a record of >80% sick leave certificate on the day of treatment. This criterion excluded patients that received disability benefits or were not in employment before treatment, and those without permanent residence permit who are not eligible to receive sickness absence compensation. Patients who died or retired within a year after treatment were considered not working in the cross-sectional data analyses and censored in the Kaplan-Meier analysis. Patients that were treated for UIA within a year from treatment for a ruptured aneurysm were also censored in the Kaplan-Meier analysis.

### Statistical analysis

Statistical analyses were performed using R: A language and environment for statistical computing, version 3.6.3 [13] and IBM SPSS Statistics for Windows, Version 32 27.0.1.0 [14]. Normal distribution of continuous variables was assessed with Q-Q plots. Differences in patient characteristics were analysed using Chi-square test for categorical variables and Mann-Whitney U test for comparison of skewed continuous variables. Patients were considered not working if they received sickness or disability benefits for more than 80% of full employment. They were considered working part-time if they received any sickness or disability benefits less or equal to 80% of full employment. Working status after treatment was compared between patients treated with endovascular treatment and surgical clipping using Kaplan-Meier analysis and the log rank test. Return to work (“event” in the analysis) was defined as the first day at work in at least 20% employment in a continuous 3-month working period. A Cox regression model assessing potential predictive factors for return to work was developed. Age at treatment was treated as a continuous variable in the model. The proportional hazards assumption was tested using the Schoenfeld residuals test.

### Ethical approval

The Regional Committee for Medical and Health Research Ethics in Central Norway approved the study (2019/521) and waived the requirement of informed consent.

## Results and discussion

**Fig 1:**
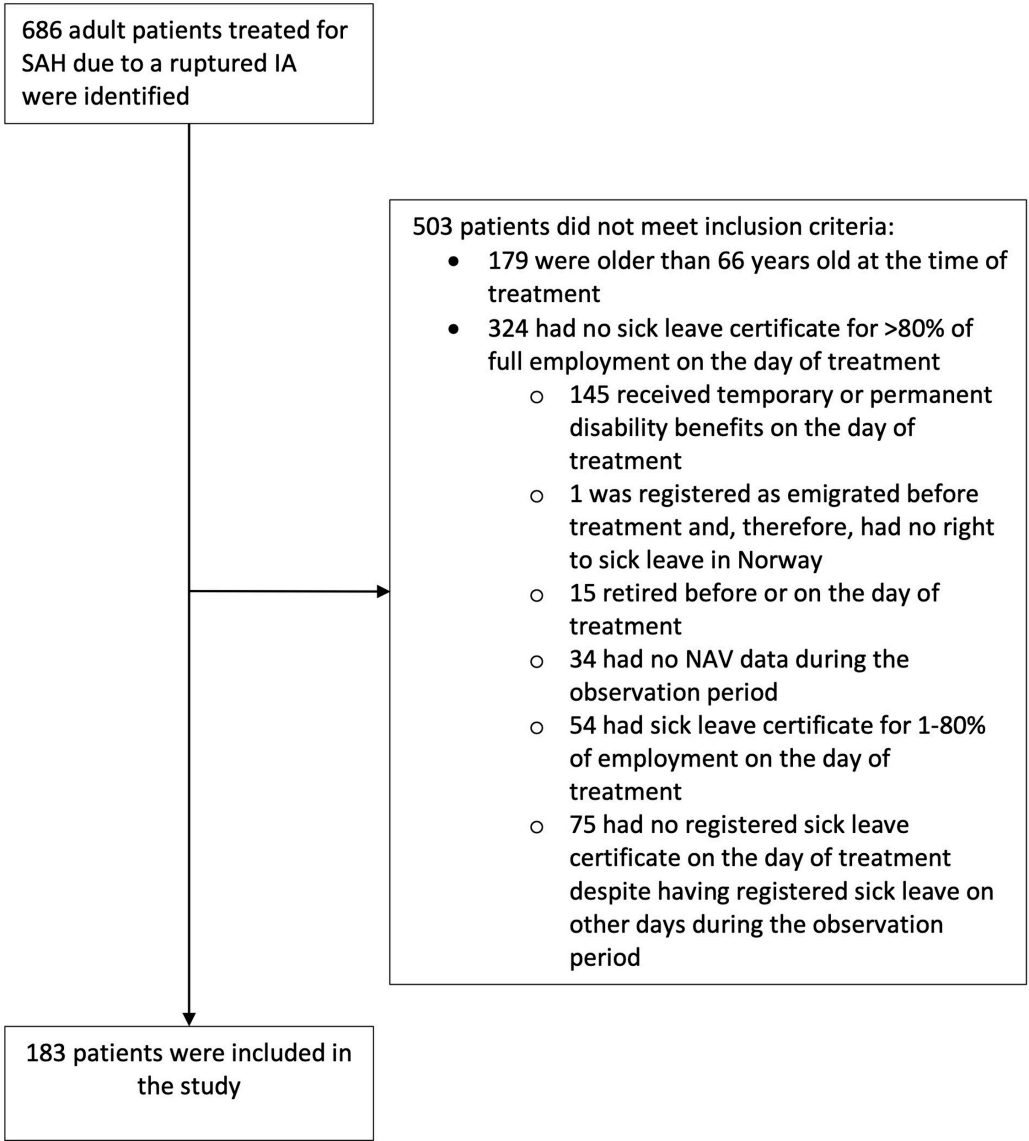
The selection of the study population.

The inclusion and exclusion process is shown in Fig 1.

Of the 183 patients included in the study, 109 (60%) were female. Median age at treatment was 51 years (range: 25–66 years). The cohort included 99 (54%) patients treated endovascularly and 84 (46%) patients who underwent open surgery. Seven patients treated initially with coiling were retreated for the same ruptured aneurysm within a year from the first treatment. Of those, 4 were treated with clipping and 2 were treated with second coiling. Four patients were retreated in the clipping cohort. Of those, 2 were treated with coiling and 2 were treated with second open surgery. Patient characteristics are shown in Table 1.

**Table 1:** Patient characteristics (n = 183).

Characteristics	Results
**Sex**	109 females/74 males
**Age at treatment (median/range)**	51 years (25–66 years)
**Treatment (endovascular/open surgery)**	99 (54%)/84 (46%)
**ICD-10 diagnosis**	
SAH from carotid artery	10 (5.5%)
SAH from middle cerebral artery	51 (27.9%)
SAH from anterior communicating artery	74 (40.4%)
SAH from posterior communicating artery	11 (6.0%)
SAH from basilar artery	15 (8.2%)
SAH from vertebral artery	3 (1.6%)
SAH from other intracranial arteries	11 (6.0%)
SAH from unspecified intracranial artery	3 (1.6%)
Other nontraumatic subarachnoid haemorrhage	2 (1.1%)
Nontraumatic subarachnoid haemorrhage, unspecified	3 (1.6%)

SAH—subarachnoid haemorrhage

**Fig 2:**
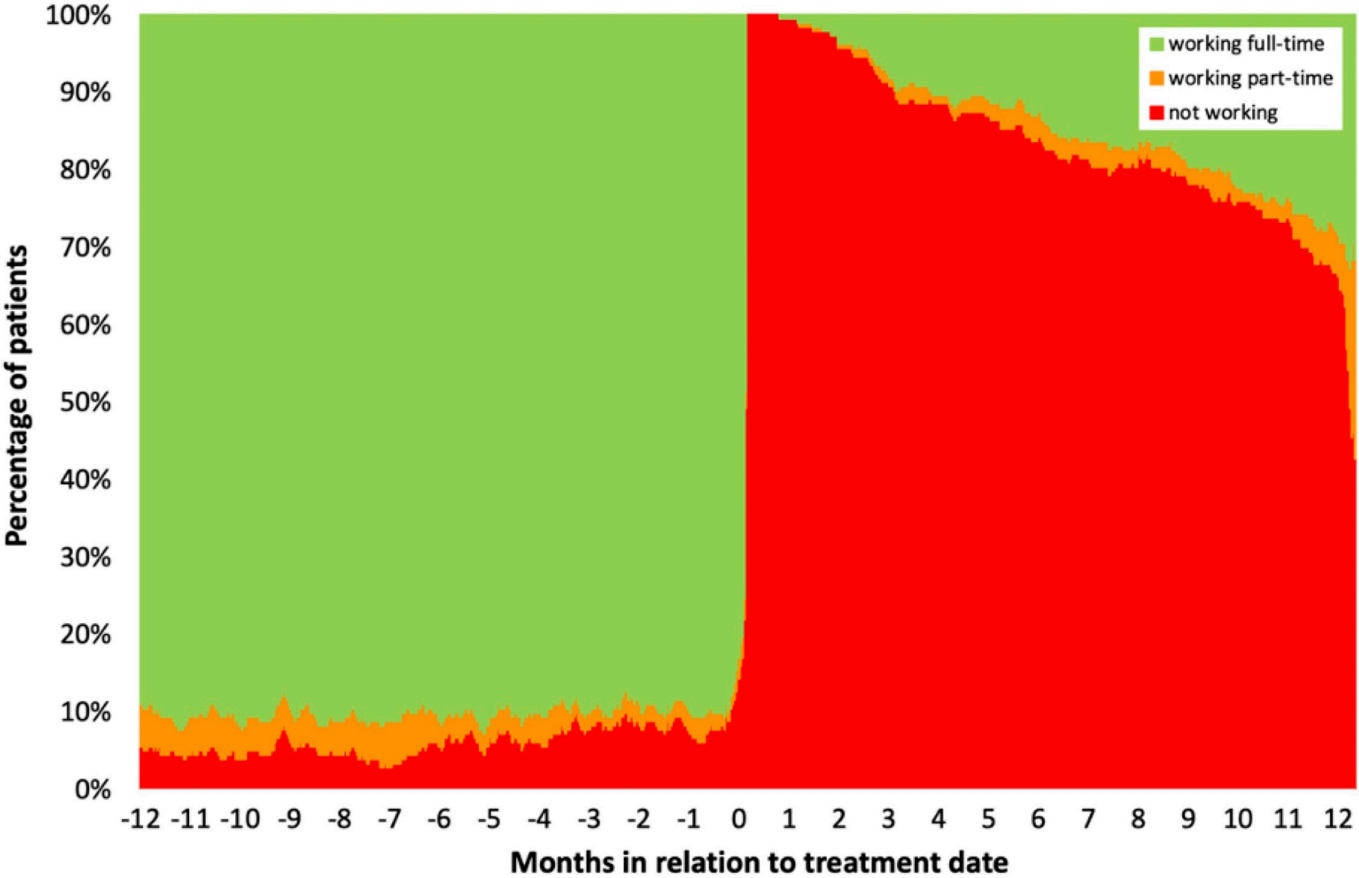
Working status before and after treatment for ruptured IAs (n = 183).

Fig 2 shows daily proportion of working status among patients treated for aSAH during a 2-year period, from one year preoperatively to one year postoperatively (n = 183). In the study population, the mortality rate was 4% and 6% at 30 days and 1 year post-treatment, respectively.

Longitudinal analysis of working status of patients who worked at one year preoperatively was performed. Among patients who were registered as working (full-time or part-time) one year preoperatively, 57% had returned to work one year after aSAH treatment. Among patients who worked full-time at one year before treatment, 33% had returned to full-time and 25% to part-time employment one year after aSAH treatment. Among patients who worked part-time at one year before treatment, 30% worked part-time and 20% worked full-time one year after the treatment for aSAH.

There was no substantial difference in patient sex (66% females vs. 52% females, *p* = 0.068) or median age at treatment (52 vs. 50 years, *p* = 0.198) between the endovascular and the surgical treatment group, respectively. There was a statistical difference in the origin of SAH between the groups (*p*<0.001). More patients with middle cerebral artery aneurysms were treated with surgical clipping while more patients with aneurysms in anterior communicating artery, posterior communicating artery, and basilar artery were treated with endovascular procedures.

**Fig 3:**
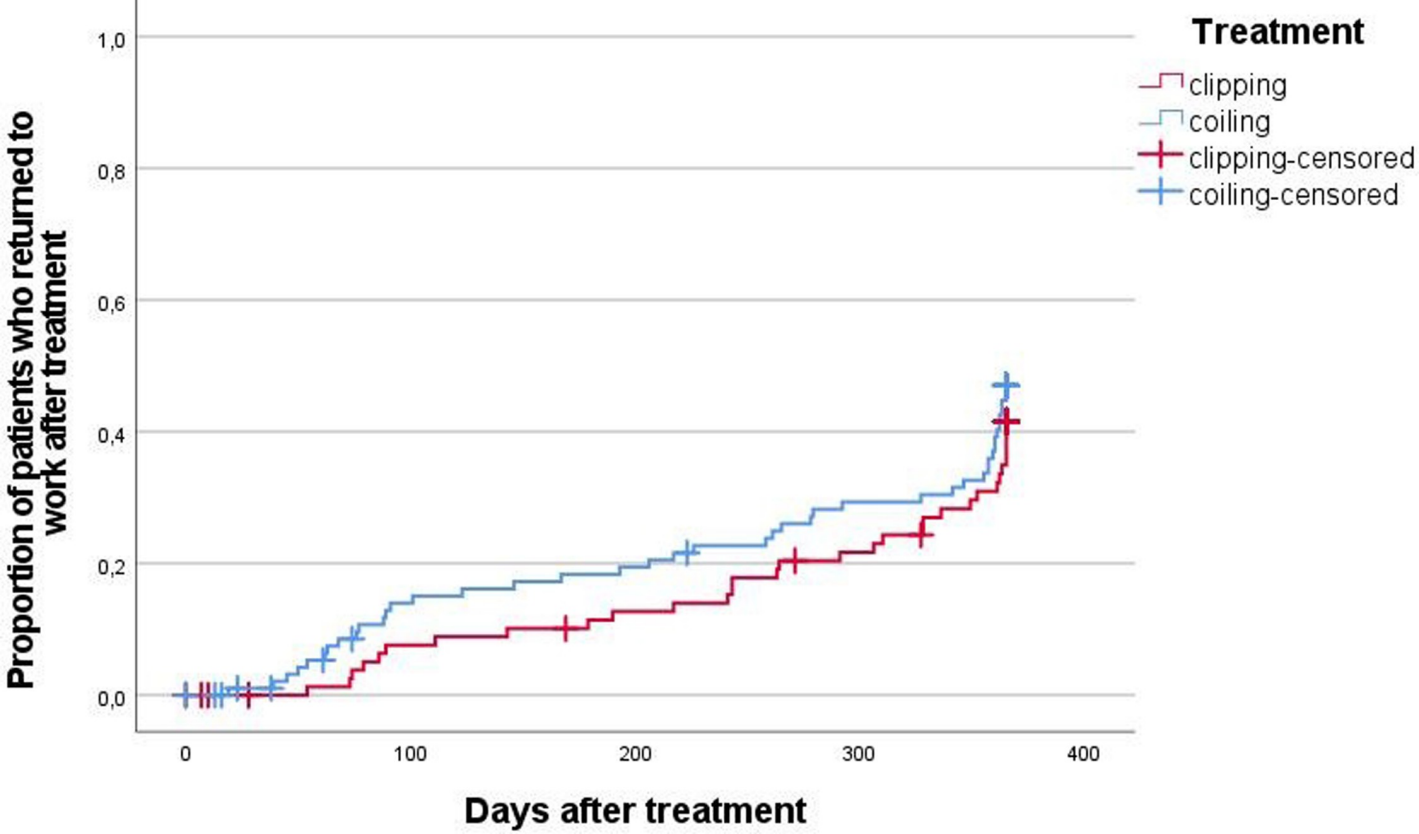
Kaplan-Meier analysis of return to work of patients treated for a ruptured IA according to treatment modality.

As shown in Fig 3, there was no significant difference in return to work after endovascular treatment versus open surgery following aSAH (*p* = 0.365). Mean number of days from treatment to the first day back at work in a continuous 3-month working period was 298 (95% CI: 276–321) vs. 319 (95% CI: 299–339) for patients who underwent endovascular treatment compared to patients treated with open surgical clipping. Eight patients treated with coiling were censored in the analysis because they died (5 patients), retired (2 patients) or were treated for another aneurysm (1 patient) within the year after treatment, before they returned to work. Nine patients treated with clipping were censored because they died (5 patients), retired (1 patient) or were treated for another aneurysm (3 patients).

A Cox regression model with age at treatment, patient sex and treatment modality was developed to investigate potential predictor factors for return to work (Table 2). Only 164 of the included patients had a defined aneurysm location (ICD-10 codes I60.0-I60.5). A separate Cox model was developed for those patients to investigate aneurysm location as a predictor factor for return to work controlled for the other factors. The multivariable analysis showed that older patients were less likely to return to work after treatment (hazard ratio 0.977, 95% CI 0.956–1.000, *p* = 0.046). There was no significant association between return to work and patient sex, treatment modality, or location of the aneurysm (anterior vs. posterior circulation).

**Table 2:** Predictor factors for return to work (n = 183).

	Univariable analysis		Multivariable analysis	
Factor	HR (95% CI)	*p*-value	HR (95% CI)	*p*-value
**Age at treatment (continuous)**	0.977 (0.955–0.999)	0.039	0.977 (0.956–1.000)	0.046
**Sex** • **Female** • **Male**	1 (reference) 1.398 (0.888–2.202)	0.148	1 (reference) 1.394 (0.880–2.208)	0.157
**Treatment modality** • **Clipping** • **Coiling**	1 (reference) 1.234 (0.781–1.950)	0.368	1 (reference) 1.328 (0.836–2.108)	0.229
Aneurysm location* • **Posterior circulation** • **Anterior circulation**	1 (reference) 1.099 (0.575–2.009)	0.776	1 (reference) 1.230 (0.627–2.412)	0.547

*164 patients were included in the analysis

In the present nationwide registry-based study work life participation in patients with ruptured intracranial aneurysms decreased after treatment. Among patients who worked one year preoperatively, only 57% returned to work one year after treatment. There was no significant difference in return to work after endovascular treatment versus open surgery following aSAH. The multivariable analysis showed that older patients were less likely to return to work after treatment. Aneurysm location was not a predictor for return to work.

Return to work after aSAH may reflect the total level of postoperative functioning in patients with good outcome. The results are of importance when informing patients and their families following aSAH. The present population-based study is the largest study to date comparing return to work following aSAH between patients treated with endovascular techniques and those treated with open surgery. Our study showed no significant difference in return to work between different treatment groups. The lack of differences can be due to the rather similar morbidity of both treatment modalities. However, the lack of differences may also reflect that the added burden of treatment, regardless of the modality, is completely overshadowed by the large impact of the subarachnoid haemorrhage itself.

Previous cross-sectional studies found that between 32–73% of patients returned to work after aSAH [15–18]. A Swedish longitudinal registry-study reported that 39% of women and 28% of men had at least one episode of sickness absence and/or disability pension during the 4^th^ year after aSAH [19]. Although a sub-analysis in one of the studies showed results in line with ours, that about 50% of patients who worked pre-haemorrhage returned to work at one year postoperatively [16], previous studies used different methods that hamper comparisons with our study. First of all, the assessment of working status was often poorly described. One of the studies did not assess the working status before aSAH and most studies had long and very variable intervals between treatment and the return to work assessment [15–18]. Only one of the mentioned studies compared return to work between patients treated with endovascular treatment and surgical clipping [18]. Similarly to our study, the authors found no significant difference in return to work between the groups [18].

The national registry based collection of data is a major strength in the present study enabling inclusion of data on all sickness absence certified by a doctor and all disability benefits received by all individuals with a Norwegian national security number treated for ruptured IAs in Norway in a 11-year period. In Norway, as in most countries, endovascular procedure is the first-line treatment that is considered for treatment of ruptured IA, Open surgical clipping is reserved for aneurysms where successful coiling is unlikely or has failed. Preoperative working status or patient’s comorbidities are not a deciding factor in the choice of treatment modality. The present study has some limitations. Findings from Norway with a public healthcare system and generous social benefits might not be directly extrapolated to other countries. Norway has a state funded public healthcare system where all permanent residents have equal access to postoperative care and rehabilitation regardless of the treatment modality used. Both treatment types are performed in the same hospitals. All aSAH patients are managed by the same neurosurgical teams pre- and postoperatively regardless of the treatment modality. Nevertheless, we believe that the burden of aSAH, as experienced by patients is still reflected in the Norwegian employment numbers postoperatively, although the number of patients returning to work may differ across nations. Our data show that many patients return to work just before one year after treatment. This could be influenced by the sickness benefit system in Norway since sickness compensation decreases significantly after one year. Although data are considered reliable because the sickness compensation is paid according to the records, the study design assumes that when there are no records of sickness or disability benefits, the individuals are in full employment. Patients that did not have a sick leave certificate for >80% of full employment on the day of treatment were excluded from the study. Most of those patients were individuals that received disability benefits. Some were retired on the day of treatment and one was registered as emigrated and had, therefore, no right to sickness benefits in Norway. 34 patients had no NAV data during the observation period. Those patients are most likely individuals without permanent resident permit and have, therefore, no right to sickness benefits in Norway. 75 patients had no registered sick leave certificate despite having registered sick leave on other days during the observation period. Those patients could have been self-employed individuals that do not receive sickness compensation from the state in Norway during the first 16 days of sickness. Moreover, this group could have also included students or unemployed individuals that are not entitled to sickness benefits. In addition, return to work in the observation period was likely to be affected not only by the aneurysm treatment, but also severity grade of aSAH (e.g. WFNS grade on admission) and patient comorbidities. These were unknown variables and could have introduced confounding to the comparative analysis. Nevertheless, patient clinical condition or comorbidities are not factors that affect the choice of treatment modality. Finally, this study included only patients that were working during the year before aSAH and untreated patients, e.g. those that died before treatment, were not included in the study. Thus, the mortality and morbidity associated with aSAH presented in this study is, therefore, underestimated and the results reflect outcome of employed aSAH patients who undergo treatment.

## Conclusions

In conclusion, aSAH profoundly affects working status. We found no difference in return to work after treatment between patients treated with endovascular techniques compared to patients undergoing open surgery. The burden of haemorrhage is likely to be far more important in those patients than the burden of treatment.
